# Exogenous endothelial cells as accelerators of hematopoietic reconstitution

**DOI:** 10.1186/1479-5876-10-231

**Published:** 2012-11-21

**Authors:** J Christopher Mizer, Thomas E Ichim, Doru T Alexandrescu, Constantin A Dasanu, Famela Ramos, Andrew Turner, Erik J Woods, Vladimir Bogin, Michael P Murphy, David Koos, Amit N Patel

**Affiliations:** 1 Regen BioPharma Inc, San Diego, CA, USA; 2 Medistem Inc, San Diego, CA, USA; 3 University of Connecticut School of Medicine, Hartford, CT, USA; 4 University of Washington, Seattle, WA, USA; 5 Cook General Biotechnology LLC, Indianapolis, IN, USA; 6 Indiana University, Indianapolis, IN, USA; 7 University of Utah, Salt Lake City, UT, USA

## Abstract

Despite the successes of recombinant hematopoietic-stimulatory factors at accelerating bone marrow reconstitution and shortening the neutropenic period post-transplantation, significant challenges remain such as cost, inability to reconstitute thrombocytic lineages, and lack of efficacy in conditions such as aplastic anemia. A possible means of accelerating hematopoietic reconstitution would be administration of cells capable of secreting hematopoietic growth factors. Advantages of this approach would include: a) ability to regulate secretion of cytokines based on biological need; b) long term, localized production of growth factors, alleviating need for systemic administration of factors that possess unintended adverse effects; and c) potential to actively repair the hematopoietic stem cell niche. Here we overview the field of hematopoietic growth factors, discuss previous experiences with mesenchymal stem cells (MSC) in accelerating hematopoiesis, and conclude by putting forth the rationale of utilizing exogenous endothelial cells as a novel cellular therapy for acceleration of hematopoietic recovery.

## Background

During hematopoietic stem cell (HSC) transplantation, the recipient is exposed to combinations of chemotherapy and/or radiotherapy that result in destruction of endogenous HSC thereby creating space in the bone marrow niche for donor cells to engraft [1,2]. Unfortunately, as a result of existing disease and due to the conditioning regimen, there is a delayed time that elapses while the donor cells are re-establishing hematopoiesis in the recipient bone marrow microenvironment. This period is associated with pancytopenia and increased risk of bacterial, fungal, and viral infection. During bone marrow or mobilized peripheral blood stem transplantation, this “risk period” is approximately three to four weeks after myeloablative transplantation [3].

One of the drawbacks of HSC transplantation is the lack of donors. Approximately 30% of patients have a related donor that can meet the stringent requirement of a 6/6 or 5/6 HLA match when HSC transplants are performed. When unrelated donors are required, it takes approximately four months to find a match using registries and minorities are almost impossible to match [4-6]. In contrast to HSC transplants, cord blood transplantation does not require stringent matching and can be performed using 4/6 or even 3/6 matching, in part due to the immature nature of the cells [7,8]. This obviously increases the donor pool available. As a result, there is an increasing use of cord blood as a source of stem cells for transplants. Cord blood is superior to bone marrow and peripheral blood HSC in terms of reduced graft versus host disease and matching ability [9-11]. The time to engraftment for cord blood, however, is approximately four to seven weeks [12]. Studies have shown that adults receiving cord blood transplants have a relatively high rate of adverse events. For example, in one study, of 68 patients with hematological malignancies, 60 patients survived 28 days or more after transplantation. Of these, 55 had neutrophil engraftment at a median of 27 days. At 22 month follow-up, 19 of the 68 patients were alive and only 18 of these were disease-free 40 months after transplantation [13]. In another study, myeloablative therapy followed by infusion of unrelated umbilical cord blood cells was performed in 57 adult patients with high-risk, hematological disease. All patients received granulocyte colony-stimulating factor (G-CSF) after transplantation until neutrophil recovery. Neutrophil recovery (neutrophil count of 500/microL) occurred at 26 days and platelet recovery (>2,0000/microL) at 84 days. The median survival of the entire group was 91 days. Of the 57 patients, 11 were alive at a median follow-up of 1,670 days with infection being the primary cause of death [14].

Thus one of the limiting factors to HSC transplantation is acceleration of engraftment of the donor cells, particularly in cord blood transplantation. In order to provide a framework for discussing means of acceleration HSC reconstitution, we will describe various hematopoietic growth factors that have entered clinical use. This will position us to make the case for utilization of various cells as therapeutics for hematopoietic reconstitution, with the concept that cells may function as “homeostatic producers” of growth factors according to the body’s needs.

### Use of hematopoietic growth factors for acceleration of engraftment/hematopoiesis

Currently, hematopoietic growth factors are used to reduce the period of cytopenia in the context of transplantation. The most widely used hematopoietic growth factor is Neupogen^TM^ (Filgastrim), which is an E coli produced form of granulocyte colony stimulating factor (G-CSF) [15]. Early, open label studies demonstrated this biological drug was capable of augmenting absolute neutrophil counts dose-dependently 1.8 – 12.0 fold in cancer patients undergoing chemotherapy [16], shortening time until neutrophil recovery post myeloablation, and shortening median febrile days and days in hospital [17]. Potency of Neupogen in non-transplant associated neutropenia was demonstrated in a double blind, placebo controlled trial, 123 patients with recurrent infections (absolute neutrophil count < 0.5 x 10^9^/L) were randomized to either receive Neupogen or enter a four month observation period followed by Neupogen administration. Blood neutrophil counts, bone marrow histology, and infection-related events were evaluated. Neupogen administration was associated with significant decreases in infection related events of approximately 50% as well as an almost 70% reduction in duration of antibiotic use [18]. Another double blind study examined 218 patients with cancer who had fever (temperature > 38.2 degrees C) and neutropenia (neutrophil count < 1.0 x 10^9^/L) after chemotherapy. Patients were randomly assigned to receive Neupogen (12 micrograms/kg of body weight per day) (n = 109) or placebo (n = 107). Patients received treatment and remained in the study until the neutrophil count was greater than 0.5 x 10^9^/L and until four days without fever (temperature < 37.5 degrees C) occurred. Compared with the placebo, Neupogen reduced the median number of days of neutropenia and the time to resolution of febrile stage but not days of fever. The frequency of the use of alternative antibiotics was similar in the two groups. The median number of days patients were hospitalized while in the study was the same (8.0 days; P = 0.09). Neupogen did decrease the risk for prolonged hospitalization by half [19].

Based on the above and numerous other studies [15,18,20-28], Neupogen was approved in the USA by the FDA for decreasing the incidence of infection as manifested by febrile neutropenia in patients with nonmyeloid malignancies receiving myelosuppressive chemotherapy drugs associated with a significant incidence of severe neutropenia with fever. It is indicated for reducing the time to neutrophil recovery and the duration of fever following induction or consolidation chemotherapy in patients with acute myeloid leukemia. In patients with bone marrow transplant, is indicated to reduce the duration of neutropenia and neutropenia-related clinical sequelae (e.g.‚ febrile neutropenia in patients with nonmyeloid malignancies undergoing myeloablative chemotherapy followed by marrow transplantation). Additionally, it is indicated for treatment of chronic neutropenia [29,30]. A delayed-release form of Neupogen, termed Neulasta (pegfilgrastin), is now also approved for similar indications by the FDA [31].

Another biological drug belonging to the HSC-stimulating family is recombinant granulocyte monocyte colony stimulating factor (GM-CSF; Sargramostim, Leukine) which was approved by the FDA for treatment of post-chemotherapy hematopoietic recovery in older adult patients with acute myelogenous leukemia (AML) to shorten time to neutrophil recovery and acceleration of myeloid reconstitution after autologous or allogeneic bone marrow transplantation (BMT). Additionally, Leukine was approved for use in bone marrow transplantation failure or engraftment delay [32]. Clinical trials with Leukine that supported its claim include a 125 patient double blind trial of post-chemotherapy AML patients in which the growth factor was given 11 days post therapy until neutrophil recovery. Time to neutrophil recovery, infectious toxicity, and treatment-related toxicity was significantly reduced in the Leukine-treated arm compared to placebo. Additionally, the median survival for patients was 10.6 months in the treated group and 4.8 months in the placebo arm [33]. Clinical trials with Leukine also included a 128 patient, multicenter, double blind trial examining neutrophil recovery after autologous bone marrow transplant. In this study, patients in the treated group had a recovery of the neutrophil count to 500 × 10^6^ per liter seven days earlier than the patients who received placebo (19 vs. 26 days), suffered from fewer infections, required 3 fewer days of antibiotic administration (24 vs. 27 days), and required six fewer days of initial hospitalization (median, 27 vs. 33 days). Unfortunately, there was no difference in survival rate at 100 days [34]. Another clinical trial with 134 patients in a double blind trial of cancer (hematological and solid) patients suffering febrile neutropenia in which Leukine enhanced neutrophil recovery [35]. The current practice is to utilize to some extent interchangeably Neuopogen and Leukine based on the patient characteristics and the familiarity of the institute.

Platelet recovery takes a longer time as compared to granulocyte recovery, during HSC reconstitution. Thrombopoietin (TPO) is a cytokine involved in stimulating megakaryocyte production from the bone marrow. In studies on small animals and non-human primates, administration of TPO resulted in a rapid rise in platelet counts to levels previously unattainable with other thrombopoietic cytokines [36]. In myelosuppressed models, use of TPO following chemotherapy, radiation, or stem-cell transplantation accelerated megakaryocyte and platelet recovery [37,38]. These studies prompted a 12 patient trial in sarcoma patients treated with a single dose of TPO. Treatment was associated with an increase in platelet counts, peaking at a 213% over baseline. This increase began by day four in most patients and peaked on a median of day 12 [39]. Other trials provided results that were somewhat inconclusive. Nash et al examined 37 patients after bone marrow transplant (both autologous and allogeneic) who suffered from thrombocytopenia that were treated with increasing doses of TPO. Ten patients had recovery of platelet counts during the 28-day study period; three of these ten had an increase in marrow megakaryocyte content seven days after completing treatment with TPO. When all baseline marrows were compared with samples after TPO treatment, there was no difference in marrow megakaryocyte content [40]. No association between dose and megakaryocyte recovery was observed. Due to further studies that demonstrated development of autoantibodies to TPO in treated patients, as well as general lack of efficacy, the clinical development of TPO was discontinued in the USA [41].

In order to overcome these potential issues associated with administration of proteins (eg sensitization), scientists started to develop agonists of the TPO receptor. One of these that was successfully developed is the TPO mimetic peptide Romiplostim (originally named AMG531) developed by Amgen. Early studies demonstrated that in vitro administration of Romiplostim binds the TPO receptor with similar affinity as TPO, induces activation of the same molecular pathways (eg Mpl, JAK2, and STAT5) and stimulates of megakaryocytic colony growth from bone marrow cells [42]. Interestingly, Amgen chose to focus clinical development of Romiplostim on immune mediated thrombocytopenia (ITP) instead of post-transplant acceleration of thrombopoiesis. Two studies demonstrated safety and improvement in platelet counts in a dose dependent manner in patients with severe ITP [43]. Another study randomized 234 adult patients with immune thrombocytopenia and provided the standard of care (77 patients) or weekly subcutaneous injections of Romiplostim (157 patients). The rate of a platelet response in the Romiplostim group was 2.3 times that in the standard-of-care group. Patients receiving Romiplostim had a significantly reduced incidence of treatment failure (18 of 157 patients [11%]) than those receiving the standard of care (23 of 77 patients [30%]). Splenectomy, a last resort treatment for ITP, also was performed less frequently in patients receiving Romiplostim (14 of 157 patients [9%]) than in those receiving the standard of care (28 of 77 patients [36%]). The Romiplostim group had a lower rate of bleeding events, fewer blood transfusions, and greater improvements in the quality of life than the standard-of-care group. Serious adverse events occurred in 23% of patients (35 of 154) receiving Romiplostim and 37% of patients (28 of 75) receiving the standard of care [44]. In August 2008, Romiplostim was approved by the FDA for the treatment of thrombocytopaenia in patients with chronic ITP who have had an insufficient response to corticosteroids, immunoglobulins, or splenectomy [45].

Current hematopoietic growth factors have several limitations: their inability to stimulate all hematopoietic lineages, their inability to heal damaged bone marrow microenvironment, and their adverse effects associated with prolonged usage. From a health economics perspective, these agents are associated with high costs. For example, according to one report, the cost of treating neutropenic fever with Neupogen is approximately $40,000 [46]. This included the cost of drug, hospital stay, and care of the patient.

### Cellular approaches for acceleration of hematopoietic reconstitution: use of mesenchymal stem cells (MSC)

It is well known that chemotherapy and radiation are damaging to the bone marrow microenvironment [47,48]. Additionally, administration of human MSC has been shown to accelerate hematopoietic reconstitution in animal models [49,50]. Although the in vivo significance of MSC is still highly debated, one theory is that MSC in the bone marrow provide a suitable environment for hematopoiesis. Accordingly, one of the first clinical uses of MSC has been to accelerate hematopoietic recovery. In a 1995 paper, Lazarus et al. reported the use of autologous, in vitro expanded, “mesenchymal progenitor cells” to treat 15 patients suffering from hematological malignancies in remission. The authors demonstrated feasibility of expanding bone marrow derived by MSC in vitro. They showed that a 10 milliliter bone marrow sample was capable of 16,000-fold growth over a four to seven week in vitro culture period. Cell administration was performed in total doses ranging from 1 - 50 × 10^6^ cells and was not causative of treatment associated adverse effects [51]. In a subsequent study from the same group in 2000, the use of MSC to accelerate hematopoietic reconstitution was performed in a group of 28 breast cancer patients who received high dose chemotherapy. MSC at concentrations of 1.0 - 2.2 × 10^6^/kg were administered intravenously. No treatment associated adverse effects where observed, and leukocytic and thrombocytic reconstitution appeared to undergo “rapid recovery” [52]. It is interesting that these initial uses were actually in patients with neoplasia and no overt acceleration of cancer progression was noted. Besides feasibility, these studies were important because they established the technique for ex vivo expansion and readministration. Despite these initial safety data, the use of mesenchymal stem cells in the context of cancer has been raised in several academic contexts. The recent regulatory approvals of the Osiris Prochymal product in Canada and New Zealand for graft versus host disease suggests that there is some degree of safety, however long term follow up studies are necessary to convincingly state that MSC grafts do not accelerate tumor formation/recurrence.

Studies along these lines continued which reaffirmed the feasibility of the approach of “repairing bone marrow stroma” with expanded MSC cells. In 2005, Lazarus et al treated 46 patients suffering from hematological malignancies with HLA-matched allografts comprising bone marrow and donor-derived expanded MSC. The numbers of MSC administered were 1 - 5 million/kg. On average, the time to neutrophil reconstitution (as defined by absolute neutrophil count > or = 0.500 × 10^9^/L) and platelet reconstitution (as defined by platelet count > or = 20 x 10^9^/L was 14.0 days (range 11.0 - 26.0 days) and 20 days (range 15.0 - 36.0 days). Incidence of acute, Grade II-IV GVHD was 13/46 and chronic was 22/36 patients that survived for at least 90 days. Relapse of malignancy occurred in 11 patients with a median time to progression of 213.5 days (range 14 - 688 days). The authors concluded that cotransplantation of HLA-identical sibling culture-expanded MSCs with an HLA-identical sibling HSC transplant was feasible and safe, without immediate infusional or late MSC-associated toxicities [53]. These data were of importance since one of the concerns regarding MSC treatment is associated with growth factor production. Leukemic patients have minimally residual disease which seems to be at least in part controlled by recipient immune function [54,55]. The demonstration that the recipients did not have an overtly higher incidence of relapse, based on clinical experiences of the authors with the specific hematological malignancies, suggests that MSC do not endow a preferential advantage to leukemic cells. This is interesting given that MSC are generally considered immune suppressive cells [56,57].

Other studies also supported the safety aspect and included several variations. For example, Ball et al reported on the use of purified, donor-specific MSC (1 - 5 million/kg) injected alongside with isolated CD34 from HLA-mismatched relatives in 14 pediatric leukemia patients. They showed that in contrast to traditional graft failure rates of 15% in 47 historical controls, all patients given MSCs showed sustained hematopoietic engraftment without any adverse reaction. Interestingly, children given MSCs did not experience more infections compared with controls [58]. Zhang et al [59] reported that 12 patients cotransplanted with donor MSC (1.77 +/- 0.40) × 10^6^/kg and HSC. No observable, adverse response during and after the infusion of MSCs was reported and hematopoietic reconstitution occurred rapidly. Two patients developed grade II-IV acute GVHD and two extensive chronic GVHD. Four patients suffered from cytomegalovirus infection but were cured ultimately. Up to the time of publication, seven patients had been followed as long as 29 - 57 months and five patients died. It was concluded by the authors that MSCs can be expanded effectively by culture and are safe and feasible to cotransplant in patients with allogenic, culture-expanded MSCs.

As mentioned above, engraftment of cord blood occurs over a more protracted time period as compared to bone marrow. Macmillan et al used parental haploidentical MSC to promote engraftment in 15 pediatric recipients of unrelated donor umbilical cord blood for acute leukemias. Eight patients received MSCs on day zero with three patients having a second dose infused on day 21. The average dose of the first infusion was 2.1 million/kg (range 0.9 –5.0/kg). The second infusion was 1 million, 600,000, and 5 million per kg. The reason for the inconsistency was lack of ability to expand cells in vitro. No serious adverse events were observed with any MSC infusion. All eight evaluable patients achieved neutrophil engraftment at a median of 19 days. Probability of platelet engraftment was 75% at a median of 53 days. At the median follow-up of 6.8 years, five patients were alive and disease free [60]. Meuleman et al used donor-derived expanded MSC (10^6^/kg) to treat six patients to accelerate hematopoietic recovery. Two patients displayed rapid hematopoietic recovery (days 12 and 21) and four patients showed no response. One patient developed cytomegalovirus (CMV) reactivation 12 days following the MSC infusion and died from CMV disease, although the authors stated that it was impossible to discern whether the reactivation was associated with the MSC therapy or prior immune suppressive regimen [61].

Use of third-party MSC to enhance peripheral blood stem cell grafts was performed by Baron et al in 20 patients who received non-myeloablative hematopoietic stem cell transplant. The outcomes were compared to a control group of 16 patients receiving a similar transplant protocol without MSC. MSC were administered one-half hour to two hours before the hematopoietic graft. Out of the 20 patients, one had primary graft failure. One year non-relapse mortality was 10% and relapse occurred in 30%. Overall survival was 80%, progression-free survival was 60%, and 1-year incidence of death from GVHD or infection with GVHD was 10%. In the control group, the one year incidence of non-relapse mortality was 37% (P = .02); the one year incidence of relapse was 25% (NS). The one year overall survival and progression free survival was 44% (P = .02), and 38% (P = .1), respectively. The one year rate of death from GVHD or infection with GVHD of 31% (P = .04) [62]. Of particular interest is that the nonmyeloablative protocol used in this study depends largely on donor graft versus leukemia effect [63]. Therefore, because the MSC did not cause a greater increase in leukemic relapse, there is suggestion that these cells may not be cancer-promoting, at least not from the perspective of immune suppressive activities. These data suggest that MSC coinfusion does not accelerate relapse may actually possess beneficial properties in terms of graft versus tumor events. There is still some controversy in that Ning et al showed that out of ten patients who received MSC coinfusion, six had relapses, whereas only three of the fifteen who received transplants without MSC had relapses [64]. There is some debate whether patient selection in the study was appropriately matched between controls and treated groups [65]. Drawbacks of using MSC for hematopoietic engraftment also include the potential risk of acceleration of tumor relapse [64]. Specifically, the immune modulatory properties of these cells may decrease the GVL effect.

Thus the use of cells to stimulate hematopoietic reconstitution appears to be clinically feasible as a possible adjuvant or replacement for hematopoietic growth factor administration. Possible advantages of using cellular based therapies as compared to individual growth factors include: a) that multiple cytokines and growth factors are produced, which may have synergistic effects not observed with single cytokine administration; b) production of factors may be regulated based on physiological and microenvironmental signals; and c) anti-inflammatory effects of cells may have additional benefits in terms of healing bone marrow microenvironment. Below we will discuss.

### Endothelial cells as a component of the hematopoietic stem cell niche

Originally thought of as an inert structure, over the past two decades the endothelium has received significant attention as a dynamic surface cell that acts as an adaptable, anticoagulated barrier between the blood stream and interior of the blood vessel. This allows for selective transmigration of cells in and out of the blood stream, regulates blood flow through controlling smooth muscle contraction, and participates in tissue remodeling and angiogenesis [66-70]. Embryonically, endothelial cells are believed to originate from a stem cell, the hemangioblast, which is capable of giving rise to both hematopoietic and endothelial cells [71]. During adulthood, the endothelium is continually self-renewed by a population of bone marrow-derived cells termed endothelial progenitor cells (EPC). This progenitor population has previously been characterized as expressing the CD34 HSC marker as well as VEGF-receptor 2 and AC133 [72]. These cells have been demonstrated using in vivo chimeric models to repair damaged blood vessels in non-diseased [73] as well as in pathological settings [74,75].

Given the importance of endothelial cells in so many aspects of biological systems, it would be reasonable to explore the ways in which endothelial cells provide support, if not control, for hematopoietic processes. Specifically, the observation that hematopoietic and endothelial cells originate developmentally from a common precursor may suggest that in adulthood these cells are inter-related. The original experiments highlighting the interaction between hematopoietic cells and endothelial cells were studies which attempted to recapitulate hematopoiesis in vitro. Early experiments demonstrated that an endothelial cell layer was essential as part of the “stroma” for in vitro hematopoiesis [76,77]. Interestingly, soluble factors generated by endothelial cells that supported in vitro hematopoiesis were not only identified [78], but it was demonstrated that their production was inducible by various agents such as lipopolysaccharide [79]. This suggests that the endothelium was an inducible source of factors stimulating hematopoiesis when physiologically necessary, such as during infection [80]. Detailed characterization of the role of endothelium in hematopoiesis was performed initially by anatomical studies which defined the sinusoidal aspects of the bone marrow hematopoietic endothelium [81,82]. Morphological changes of this specialized endothelium have been identified during times of excessive production of various blood cells [83], hinting at a possible involvement in the process of hematopoiesis [84]. During times of inflammation, structural changes occur in the bone marrow endothelium, in part to support release of granulocytes [85]. Such changes also occur in response to administration of bone marrow stem cell mobilizing agents, such as Neupogen, which causes stimulation of localized complement activation through activation of proteases that expose neoepitopes in the bone marrow microenvironment [86,87]. Because of the physical proximity of HSC and endothelial cells, as well as the ability of endothelial cells in vitro to support hematopoiesis, investigators have sought to identify molecular means by which endothelial cells may control hematopoiesis.

### Endothelial cells produce hematopoietic growth factors

The factors controlling hematopoiesis were originally described by biological activity in colony forming assays before the era of molecular biology unfolded. Knudtzon and Mortensen in 1975 described experiments in which bone marrow mononuclear cells were plated on agar and colony formation was assessed as a means of quantifying stem cell proliferation and differentiation. They observed that human endothelial cells were capable of specifically stimulating granulocyte colonies. Specifically, they co-cultured vein fragments or isolated endothelial cells separated from the vein of human umbilical cords. The granulocyte colony stimulating activity was superior to that obtained by co-culture with leukocytes. They suggested that in addition to monocytes, that bone marrow resident endothelial cells supported proliferation and granulocytic differentiation of hematopoietic stem cells [88].

Five years later, Quesenberry and Gimbrone used a similar assay system and observed not only that endothelial cells were capable of stimulating hematopoiesis and granulopoiesis, but also that pretreatment of the endothelial cells with either endotoxin or granulocytes augmented the ability of endothelial cells to promote granulopoiesis [79]. Further studies revealed that treatment of endothelial cells with TNF-alpha, a potent mediator of inflammation, actually resulted in augmentation of hematopoietic stimulatory activity, in part through production of a protein that was later identified as GM-CSF [89]. A similar finding was demonstrated with the inflammatory cytokine IL-1 [90].

Ascensao et al sought to identify other mechanisms responsible for endothelial cell stimulation of bone marrow hematopoiesis. They identified not only the stimulation of granulopoiesis but also erythropoiesis. Specifically, they found that endothelial cells produce proteins of approximately 30,000 Daltons with isoelectric focusing points of 4.5 and 7.2 that stimulated the growth of human BFU-E and CFU-mix. They also found heat-labile proteins of 30,000 and 15,000 Daltons inducing the proliferation and differentiation of granulocyte-macrophage (CFU-GM) colonies. Interestingly, no erythropoietin was found in the culture [91]. More detailed examination revealed that GM-CSF was one of these proteins [92]. Several other studies have confirmed GM-CSF production from endothelial cells. Malone et al used adipose derived endothelial cells and demonstrated GM-CSF production in an unstimulated state [93]. A table describing some of the hematopoietic growth factors produced by endothelial cells both under stimulated and unstimulated conditions is provided below (Table 1).

**Table 1:** Hematopoietic factors produced by endothelial cells

**Hematopoietic factors produced by endothelial cells**			
**Source of endothelium**	**Stimulant**	**Cytokine/CSA**	**Reference**
Umbilical vein and HUVEC	None	Granulocyte stimulatory activity	[88]
HUVEC	Endotoxin and granulocyte contact	Granulocyte-monocyte colony stimulating activity	[79]
HUVEC	None	Granulocyte-monocyte, and erythroid colony stimulating activity	[91]
HUVEC	Muramyl peptides	Granulocyte-monocyte colony stimulating activity	[94]
HUVEC	TNF-alpha	Granulocyte-monocyte colony stimulating activity	[89]
Primary and immortalized HUVEC	IL-1	Granulocyte-monocyte colony stimulating activity	[90]
Immortalized HUVEC	None	Granulocyte-monocyte colony stimulating activity	[92]
Fat capillary endothelial cells	None	GM-CSF	[93]
HUVEC	IL-1	Erythrocyte colony stimulating activity	[95]
HUVEC	Poly (IC)	Monocyte, Granulocyte, Granulocyte-Monocyte stimulatory activity	[96]
Brain perivascular endothelial cells	None	M-CSF	[97]
HUVEC	None	Stem Cell Factor	[98]
HUVEC versus bone marrow microvessels	None	High expression of heparan sulfate proteoglycans only on bone marrow microvessels, these present SDF-1	[99]
HUVEC	None	IL-3, IL-6, Stem Cell Factor	[100]
HUVEC	IL-1	GM-CSF, G-CSF	[101]
HUVEC	None	IL-11	[102]

### Endothelial cells stimulate hematopoiesis

One of the major goals of hematology research has been the development of methods of expanding hematopoietic stem cells outside of the body. This would hypothetically allow for various approaches to purging leukemic cells out of autologous grafts while expanding non-malignant hematopoietic cells, as well as expanding cord blood stem cells in order to accelerate engraftment after transplantation [103-105]. As previously discussed, early studies have used bone marrow stromal cells for expanding hematopoietic stem cells [106]. Components of the bone marrow stroma include monocytes, adipocytes, and mesenchymal stem cells [107]. An interesting finding was that endothelial cells, whether originating from bone marrow, brain, or fat, all possessed the ability to stimulate hematopoietic cell expansion. Specifically, Davis et al [108] examined the ability of porcine microvascular endothelial cells (PMVECs) together with combinations of cytokines (GM-CSF, IL-3, SCF, IL-6) to support the expansion and development of purified human CD34+ bone marrow cells. In seven day cultures, the greatest HSC expansion was observed when the HSC were in direct contact with PMVEC monolayers, followed by PMVEC noncontact and liquid suspension cultures. Maximal expansion of nonadherent cells (42-fold) and total CD34+ cells (12.6-fold) occurred in PMVEC contact cultures treated with GM-CSF + IL-3 + SCF + IL-6, with similar increases in the number of granulocyte-macrophage colony-forming units (CFU-GM), CFU-mix, erythroid burst-forming units (BFU-E), CFU-blast and CFU-megakaryocyte (CFU-Mk) progenitor cells. In long-term PMVEC contact cultures, CD34+ cells seeded onto PMVEC monolayers with GM-CSF + IL-3 + SCF + IL-6 showed a total calculated expansion of over 5,000,000-fold of nonadherent cells over 35 days in culture. These experiments not only demonstrated efficacy of endothelial cells at stimulating HSC proliferation, but also that such factors are not species-specific, thus enabling animal experiments with human cells. These studies were also confirmed in another paper [109].

Other studies have associated endothelial cell production of IL-6, SCF, G-CSF and GM-CSF ex vivo HSC expansion [110]. The expansion of HSC by endothelial cells has been shown to accelerate bone marrow engraftment in vivo [111]. Acceleration of hematopoietic recovery has been demonstrated not only with endothelial cells but also with conditioned media of these cells, suggesting both contact dependent and contact independent effects [112]. Ex vivo expansion of HSC using endothelial cells was demonstrated to generate HSC that were functional in a large animal model [113], specifically, a control baboon received no transplant and two animals that received a suboptimal number of marrow mononuclear cells died 37, 43, and 59 days post-irradiation. Immunomagnetically selected CD34(+) marrow cells from two baboons were placed in porcine microvascular endothelial cells (PMVEC) co-culture with exogenous human cytokines. After 10 days of expansion, the grafts represented a 14-fold to 22-fold increase in cell number, a 4-fold to 5-fold expansion of CD34(+) cells, a 3-fold to 4-fold increase of colony-forming unit-granulocyte-macrophage (CFU-GM), and a 12-fold to 17-fold increase of cobblestone area-forming cells (CAFC) over input. Both baboons receiving the treatment became transfusion independent by day 23 post-transplant and achieved absolute neutrophil count ANC >500/microL by day 25 ± 1 and platelets > 20,000/microL by day 29 ± 2.

In addition to expanding functional HSC ex vivo, the direct injection of exogenous endothelial cells has demonstrated hematopoietic stimulatory effects. Salter et al treated BALB/c mice with total body irradiation (TBI) followed by infusion with C57Bl6-derived endothelial progenitor cells (EPCs). TBI caused pronounced disruption of the BM vasculature, BM hypocellularity, ablation of HSCs, and pancytopenia in control mice. Irradiated, EPC-treated mice displayed accelerated recovery of BM sinusoidal vessels, BM cellularity, peripheral blood white blood cells (WBCs), neutrophils, platelets, and a 4.4-fold increase in BM HSCs. Systemic administration of anti-VE-cadherin antibody significantly delayed hematologic recovery in both EPC-treated mice and irradiated, non-EPC-treated mice compared with irradiated controls [114]. Such hematopoietic stimulatory effects were observed with endothelial cells from a variety of sources. For example, it has been shown that both brain and fetal blood derived endothelial cells have ability to endow radioprotection, as well as accelerate reconstitution in C57Bl6 mice treated with 1050 cGy of radiation [115]. In another series of experiments, Fleming’s group demonstrated that transplantation of segments of adult thoracic aorta or inferior vena cava under the kidney capsule of lethally irradiated recipients (1100 cGy) resulted in significant radioprotection. Specifically, 10 mg of transplanted vascular tissue would protect 80% of recipients from lethality. Furthermore, this procedure gave rise to similar numbers of colony forming units as rescue using 10^5^ bone marrow cells and prevented the development of severe anemia. Labeling of proliferating cells using BRDU revealed that the cells within the intima of donor vascular tissue would begin proliferation within 48 hours of transplantation. It was also demonstrated in cell tracking studies that donor-derived vascular cells migrated to the recipient spleen; hematopoietic colony forming units were of host origin. Although donor-derived cells were readily detected in the peripheral blood two to three weeks after transplant, they rapidly declined in frequency to approximately 1.0% by four weeks and persisted at these levels for more than one year. Bone marrow from rescued primary recipients provided radioprotection after transplantation into secondary recipients, but only CD3(+) donor-derived cells were detected [116]. The question remained from these experiment about whether similar hematopoietic effects could be observed by endothelial cells alone or if they require other vascular cells that mediate therapeutic effects. This was addressed in another paper by the same group which assessed vascular endothelial cells administered in suspension. The investigators found that as little as 10^4^ endothelial cells either isolated from the murine lung or brain were capable of radioprotecting mice [117]. Furthermore, endothelial cell administration was associated with reconstitution of host hematopoiesis, with the expanded cells being able to transfer hematopoiesis to secondary recipients.

These data suggest: a) allogeneic endothelial cells, apparently regardless of source, may conceptually be useful for acceleration of hematopoietic reconstitution; b) endothelial cells actively contributed to repair of the bone marrow microenvironment; and c) cell to cell interactions mediated by specific adhesion molecules are essential for therapeutic effects. Such hematopoietic stimulatory effects were observed with endothelial cells from a variety of sources.

### Practical use of endothelial cells for stimulation of hematopoietic recovery: hemaxellerate autologous adipose derived cells as a source of endothelial cells

The most practical embodiment of the concept that endothelial cells stimulate hematopoiesis in vivo would be the utilization of autologous, adipose derived, endothelial cells. In this system, it may be useful to utilize the whole stromal vascular fraction (SVF) as a heterogenous cellular product. SVF is comprised of the mononuclear cells derived from adipose tissue and are known to contain not only endothelial cells, but also T regulatory cells, monocytes, and hematopoietic stem cells. This term is more than four decades old and is used to describe the mitotically active source of adipocyte precursors [118,119]. SVF as a source of stem cells was first described by Zuk et al who identified mesenchymal-like stem cells cells in SVF that could be induced to differentiate into adipogenic, chondrogenic, myogenic, and osteogenic lineages [120]. Subsequent to the initial description, the same group reported after in vitro expansion the SVF derived cells had surface marker expression similar to bone marrow derived MSC, comprising of positive for CD29, CD44, CD71, CD90, CD105/SH2, and SH3 and lacking CD31, CD34, and CD45 expression [121,122].

Currently liposuction is performed routinely and numerous protocols exist for autologous collection and administration of SVF which contains all of the cellular elements mentioned. In fact, safety of this procedure has been previously published for rheumatoid arthritis in a 13 patient study in which patients were monitored for over one year [123]. Several other studies have demonstrated a high content of EPC in adipose tissue [124,125]. Functional demonstration of adipose EPC was performed in experiments in which SVF was purified for CD34 positive cells. This fraction was demonstrated to induce angiogenesis in immune compromised mice that were subjected to hindlimb ischemia. Mechanistically, the cells were identified as EPC based on ability to form endothelial colonies when cultured in vitro [126]. Numerous groups have reported SVF contains cellular activity stimulatory of angiogenesis. For example, Sumi et al showed that administration of SVF but not adipocytes led to revascularization in the hindlimb ischemia model [127]. Other studies have shown that EPC-like activities are found in SVF [128], and also that conditioned media from SVF is capable of stimulating host angiogenesis [129,130]. It is reported that EPC in the SVF capable of stimulating angiogenesis directly or through release of growth factors such as IGF-1, HGF-1 and VEGF [128,130-132].

Use of non-expanded SVF is commonplace in veterinary medicine and has been shown to be safe and effective. Pioneered by the Harman group at Vet-Stem, Double blind studies of SVF for canine osteoarthritis have shown statistically significant improvements in lameness, range of motion, and overall quality of life after treatment with autologous SVF [133,134]. To date Vet-Stem has treated over 5,000 canines using local, intra-articular administration as well as systemic intravenous administration of these cells. Additionally, it is reported that over 3,000 horses with various cartilage and bone injuries have been treated with autologous lipoaspirate fractions without cellular expansion [135]. These studies have not only demonstrated safety and in some cases efficacy, but also have established the practical foundation of commercialized stem cell therapeutics, at least in the area of veterinary medicine [136]. For clinical use closed system point-of-care devices have been developed by the companies Cytori and Tissue Genesis to allow for rapid processing of adipose tissue, without need for a Good Manufacturing Practices compliant laboratory [137,138].

In the published literature, the clinical use of systemically administered SVF cells has been reported in three studies. The first study was a description of 3 patients suffering from multiple sclerosis who received intravenous administration of autologous adipose SVF. All 3 patients reported significant improvement neurologically and demonstrated a good safety profile [139]. In another paper, a single patient case report described a remission of rheumatoid arthritis [140]. More recently, a paper demonstrating one year safety of rheumatoid arthritis patients treated with autologous SVF was published [123]. The general safety of fat grafting is widely accepted in that this is a common procedure in cosmetic surgery [141,142].

The HemXellerate product is an optimized autologous SVF preparation for stimulation of hematopoiesis that is covered by one issued patent and two patent applications. In practice the physician is shipped a kit, which is used to collect adipose tissue, tissue is sent to a central processing facility, and a standardized cellular product is delivered in a ready-to-use manner. Currently the company is developing various uses for the HemXellerate production in conditions involving suppressed hematopoiesis.

## Conclusion

While significant advances have been made in the use of growth factors for stimulation of hematopoietic reconstitution, overall efficacy of this approach is limited. Cell therapy offers the possibility of administering an “intelligent” therapeutic which addresses the hematopoietic needs of the host in real-time. Autologous SVF therapy, such as the HemXellerate product, offers the possibility of delivering a heterogenous population of endothelial cells, mesenchymal stem cells, and other potential cells of interest. Given the established safety and ease of autologous SVF administration, it is anticipated that the proposed procedure will be rapidly adopted by the market.

## Competing interests

TEI and VB are shareholders of the company Medistem (MEDS) which is developing universal donor stem cell technologies. TEI, JCM, DK and VB are shareholders of the company Regen BioPharma (subsidiary of Bio-Matrix Scientific Corp (BMSN), which is developing the HemaXellerate product.

## Authors’ contributions

JCM, TEI, DTA, CAD, FR, AT, EJW, VB, MPM, DK, and ANP reviewed literature, wrote the paper, and all read the paper before submission. All authors read and approved the final manuscript.

## References

[B1] Shizuru JA Transplantation of purified hematopoietic stem cells: requirements for overcoming the barriers of allogeneic engraftment Biology of blood and marrow transplantation: journal of the American Society for Blood and Marrow Transplantation 1996 2 1 3 14 9078349

[B2] Czechowicz A Efficient transplantation via antibody-based clearance of hematopoietic stem cell niches Science 2007 318 5854 1296 9 10.1126/science.1149726 18033883 PMC2527021

[B3] Abrahamsen IW Immune reconstitution after allogeneic stem cell transplantation: the impact of stem cell source and graft-versus-host disease Haematologica 2005 90 1 86 93 15642674

[B4] Barker JN Wagner JE Umbilical-cord blood transplantation for the treatment of cancer Nat Rev Cancer 2003 3 7 526 32 10.1038/nrc1125 12835672

[B5] Beatty PG Mori M Milford E Impact of racial genetic polymorphism on the probability of finding an HLA-matched donor Transplantation 1995 60 8 778 83 7482734

[B6] Hansen JA Hematopoietic stem cell transplants from unrelated donors Immunol Rev 1997 157 141 151 10.1111/j.1600-065X.1997.tb00979.x 9255627

[B7] Kurtzberg J Results of the Cord Blood Transplantation Study (COBLT): clinical outcomes of unrelated donor umbilical cord blood transplantation in pediatric patients with hematologic malignancies Blood 2008 112 10 4318 27 10.1182/blood-2007-06-098020 18723429 PMC2581998

[B8] Yu LC Unrelated cord blood transplant experience by the pediatric blood and marrow transplant consortium Pediatr Hematol Oncol 2001 18 4 235 45 10.1080/088800101750238531 11400647

[B9] Wagner JE Gluckman E Umbilical cord blood transplantation: the first 20 years Semin Hematol 2010 47 1 3 12 10.1053/j.seminhematol.2009.10.011 20109607

[B10] Smith AR Wagner JE Alternative haematopoietic stem cell sources for transplantation: place of umbilical cord blood Br J Haematol 2009 147 2 246 61 10.1111/j.1365-2141.2009.07828.x 19796274 PMC2782564

[B11] Wagner JE Umbilical cord blood transplantation Cancer Treat Res 2009 144 233 255 10.1007/978-0-387-78580-6_10 19779887

[B12] Brunstein CG Umbilical cord blood transplantation for the treatment of hematologic malignancies Cancer control: journal of the Moffitt Cancer Center 2011 18 4 222 36 21976241 10.1177/107327481101800403

[B13] Laughlin MJ Hematopoietic engraftment and survival in adult recipients of umbilical-cord blood from unrelated donors N Eng J Med 2001 344 24 1815 22 10.1056/NEJM200106143442402 11407342

[B14] Long GD *Unrelated umbilical cord blood transplantation in adult patients*. Biology of blood and marrow transplantationjournal of the American Society for Blood and Marrow Transplantation 2003 9 12 772 780 10.1016/j.bbmt.2003.08.007 14677117

[B15] Souza LM Recombinant human granulocyte colony-stimulating factor: effects on normal and leukemic myeloid cells Science 1986 232 4746 61 5 10.1126/science.2420009 2420009

[B16] Gabrilove JL Phase I study of granulocyte colony-stimulating factor in patients with transitional cell carcinoma of the urothelium J Clin Invest 1988 82 4 1454 61 10.1172/JCI113751 2459163 PMC442704

[B17] Kennedy MJ Administration of human recombinant granulocyte colony-stimulating factor (filgrastim) accelerates granulocyte recovery following high-dose chemotherapy and autologous marrow transplantation with 4-hydroperoxycyclophosphamide-purged marrow in women with metastatic breast cancer Cancer Res 1993 53 22 5424 8 7693341

[B18] Dale DC A randomized controlled phase III trial of recombinant human granulocyte colony-stimulating factor (filgrastim) for treatment of severe chronic neutropenia Blood 1993 81 10 2496 502 8490166 PMC4120868

[B19] Maher DW Filgrastim in patients with chemotherapy-induced febrile neutropenia. A double-blind, placebo-controlled trial Ann Intern Med 1994 12 7 492 501 7520676 10.7326/0003-4819-121-7-199410010-00004

[B20] Zsebo KM Recombinant human granulocyte colony stimulating factor: molecular and biological characterization Immunobiology 1986 172 3–5 175 84 3492428 10.1016/S0171-2985(86)80097-3

[B21] Welte K Recombinant human granulocyte colony-stimulating factor. Effects on hematopoiesis in normal and cyclophosphamide-treated primates J Exp Med 1987 165 4 941 948 10.1084/jem.165.4.941 3494094 PMC2188574

[B22] Duhrsen U Effects of recombinant human granulocyte colony-stimulating factor on hematopoietic progenitor cells in cancer patients Blood 1988 72 6 2074 81 3264199

[B23] Weisbart RH GM-CSF induces human neutrophil IgA-mediated phagocytosis by an IgA Fc receptor activation mechanism Nature 1988 332 6165 647 8 10.1038/332647a0 2451784

[B24] Glaspy JA Therapy for neutropenia in hairy cell leukemia with recombinant human granulocyte colony-stimulating factor Ann Intern Med 1988 109 10 789 95 2461131 10.7326/0003-4819-109-10-789

[B25] Yuo A Recombinant human granulocyte colony-stimulating factor as an activator of human granulocytes: potentiation of responses triggered by receptor-mediated agonists and stimulation of C3bi receptor expression and adherence Blood 1989 74 6 2144 9 2553162

[B26] Neidhart J Granulocyte colony-stimulating factor stimulates recovery of granulocytes in patients receiving dose-intensive chemotherapy without bone marrow transplantation Journal of clinical oncology: official journal of the American Society of Clinical Oncology 1989 7 11 1685 92 2478670 10.1200/JCO.1989.7.11.1685

[B27] Bronchud MH The use of granulocyte colony-stimulating factor to increase the intensity of treatment with doxorubicin in patients with advanced breast and ovarian cancer Br J Cancer 1989 60 1 121 5 10.1038/bjc.1989.234 2478178 PMC2247341

[B28] Heil G A randomized, double-blind, placebo-controlled, phase III study of filgrastim in remission induction and consolidation therapy for adults with de novo acute myeloid leukemia The International Acute Myeloid Leukemia Study Group. Blood 1997 90 12 4710 8 9389686

[B29] Glaspy JA Hematopoietic management in oncology practice. Part 1. Myeloid growth factors Oncology 2003 17 11 1593 603 14682110

[B30] Cappozzo C Optimal use of granulocyte-colony-stimulating factor in patients with cancer who are at risk for chemotherapy-induced neutropenia Oncol Nurs Forum 2004 31 3 569 76 10.1188/04.ONF.569-576 15146222

[B31] Hussar DA New drugs 2003, part II Nursing 2003 33 7 57 62 quiz 63-4 10.1097/00152193-200307000-00043 12851504

[B32] Crawford J Myeloid growth factors Journal of the National Comprehensive Cancer Network: JNCCN 2009 7 1 64 83 19176207 10.6004/jnccn.2009.0006

[B33] Rowe JM A randomized placebo-controlled phase III study of granulocyte-macrophage colony-stimulating factor in adult patients (> 55 to 70 years of age) with acute myelogenous leukemia: a study of the Eastern Cooperative Oncology Group (E1490) Blood 1995 86 2 457 62 7605984

[B34] Nemunaitis J Recombinant granulocyte-macrophage colony-stimulating factor after autologous bone marrow transplantation for lymphoid cancer N Eng J Med 1991 324 25 1773 8 10.1056/NEJM199106203242504 1903847

[B35] Vellenga E Randomized placebo-controlled trial of granulocyte-macrophage colony-stimulating factor in patients with chemotherapy-related febrile neutropenia Journal of clinical oncology: official journal of the American Society of Clinical Oncology 1996 14 2 619 27 8636779 10.1200/JCO.1996.14.2.619

[B36] Prow D Vadhan-Raj S Thrombopoietin: biology and potential clinical applications Oncology 1998 12 11 1597 1604 1607-8; discussion 1611-4 9834938

[B37] Grossmann A Thrombopoietin accelerates platelet, red blood cell, and neutrophil recovery in myelosuppressed mice Exp Hematol 1996 24 10 1238 46 8765500

[B38] Fibbe WE Accelerated reconstitution of platelets and erythrocytes after syngeneic transplantation of bone marrow cells derived from thrombopoietin pretreated donor mice Blood 1995 86 9 3308 13 7579432

[B39] Vadhan-Raj S Stimulation of megakaryocyte and platelet production by a single dose of recombinant human thrombopoietin in patients with cancer Ann Intern Med 1997 126 9 673 81 9139552 10.7326/0003-4819-126-9-199705010-00001

[B40] Nash RA A phase I trial of recombinant human thrombopoietin in patients with delayed platelet recovery after hematopoietic stem cell transplantation Biology of blood and marrow transplantation: journal of the American Society for Blood and Marrow Transplantation 2000 6 1 25 34 10707996 10.1016/s1083-8791(00)70049-8

[B41] Vadhan-Raj S Clinical experience with recombinant human thrombopoietin in chemotherapy-induced thrombocytopenia Semin Hematol 2000 37 2 Suppl 4 28 34 10831286 10.1053/shem.2000.7391

[B42] Broudy VC Lin NL AMG531 stimulates megakaryopoiesis in vitro by binding to Mpl Cytokine 2004 25 2 52 60 10.1016/j.cyto.2003.05.001 14693160

[B43] Bussel JB AMG 531, a thrombopoiesis-stimulating protein, for chronic ITP N Eng J Med 2006 355 16 1672 81 10.1056/NEJMoa054626 17050891

[B44] Kuter DJ Romiplostim or standard of care in patients with immune thrombocytopenia N Eng J Med 2010 363 20 1889 99 10.1056/NEJMoa1002625 21067381

[B45] Cines DB Yasothan U Kirkpatrick P Romiplostim Nat Rev Drug Discov 2008 7 11 887 8 10.1038/nrd2741 18974747

[B46] Messori A Trippoli S Tendi E G-CSF for the prophylaxis of neutropenic fever in patients with small cell lung cancer receiving myelosuppressive antineoplastic chemotherapy: meta-analysis and pharmacoeconomic evaluation J Clin Pharm Ther 1996 21 2 57 63 10.1111/j.1365-2710.1996.tb00001.x 8809640

[B47] Galotto M Stromal damage as consequence of high-dose chemo/radiotherapy in bone marrow transplant recipients Exp Hematol 1999 27 9 1460 6 10.1016/S0301-472X(99)00076-4 10480437

[B48] Banfi A Bone marrow stromal damage after chemo/radiotherapy: occurrence, consequences and possibilities of treatment Leuk Lymphoma 2001 42 5 863 70 10.3109/10428190109097705 11697641

[B49] Almeida-Porada G Cotransplantation of human stromal cell progenitors into preimmune fetal sheep results in early appearance of human donor cells in circulation and boosts cell levels in bone marrow at later time points after transplantation Blood 2000 95 11 3620 7 10828053

[B50] Noort WA Mesenchymal stem cells promote engraftment of human umbilical cord blood-derived CD34(+) cells in NOD/SCID mice Exp Hematol 2002 30 8 870 8 10.1016/S0301-472X(02)00820-2 12160838

[B51] Lazarus HM Ex vivo expansion and subsequent infusion of human bone marrow-derived stromal progenitor cells (mesenchymal progenitor cells): implications for therapeutic use Bone Marrow Transplant 1995 16 4 557 64 8528172

[B52] Koc ON Rapid hematopoietic recovery after coinfusion of autologous-blood stem cells and culture-expanded marrow mesenchymal stem cells in advanced breast cancer patients receiving high-dose chemotherapy Journal of clinical oncology: official journal of the American Society of Clinical Oncology 2000 18 2 307 16 10637244 10.1200/JCO.2000.18.2.307

[B53] Lazarus HM Cotransplantation of HLA-identical sibling culture-expanded mesenchymal stem cells and hematopoietic stem cells in hematologic malignancy patients Biology of blood and marrow transplantation: journal of the American Society for Blood and Marrow Transplantation 2005 11 5 389 98 15846293 10.1016/j.bbmt.2005.02.001

[B54] Oka T *Evidence for specific immune response against P210 BCR-ABL in long-term remission CML patients treated with interferon*. Leukemia: official journal of the Leukemia Society of America Leukemia Research Fund, U.K 1998 12 2 155 163 10.1038/sj.leu.2400919 9519777

[B55] Pulsipher MA Allogeneic transplantation for pediatric acute lymphoblastic leukemia: the emerging role of peritransplantation minimal residual disease/chimerism monitoring and novel chemotherapeutic, molecular, and immune approaches aimed at preventing relapse Biology of blood and marrow transplantation: journal of the American Society for Blood and Marrow Transplantation 2009 15 1 Suppl 62 71 19147081 10.1016/j.bbmt.2008.11.009

[B56] Griffin MD Ritter T Mahon BP Immunological aspects of allogeneic mesenchymal stem cell therapies Hum Gene Ther 2010 21 12 1641 55 10.1089/hum.2010.156 20718666

[B57] Xue Q The negative co-signaling molecule b7-h4 is expressed by human bone marrow-derived mesenchymal stem cells and mediates its T-cell modulatory activity Stem Cells Dev 2010 19 1 27 38 10.1089/scd.2009.0076 19788399

[B58] Ball LM Cotransplantation of ex vivo expanded mesenchymal stem cells accelerates lymphocyte recovery and may reduce the risk of graft failure in haploidentical hematopoietic stem-cell transplantation Blood 2007 110 7 2764 7 10.1182/blood-2007-04-087056 17638847

[B59] Zhang X Cotransplantation of HLA-identical mesenchymal stem cells and hematopoietic stem cells in Chinese patients with hematologic diseases Int J Lab Hematol 2010 32 2 256 64 10.1111/j.1751-553X.2009.01181.x 19656235

[B60] Macmillan ML Transplantation of ex-vivo culture-expanded parental haploidentical mesenchymal stem cells to promote engraftment in pediatric recipients of unrelated donor umbilical cord blood: results of a phase I-II clinical trial Bone Marrow Transplant 2009 43 6 447 54 10.1038/bmt.2008.348 18955980

[B61] Meuleman N Infusion of mesenchymal stromal cells can aid hematopoietic recovery following allogeneic hematopoietic stem cell myeloablative transplant: a pilot study Stem Cells Dev 2009 18 9 1247 52 10.1089/scd.2009.0029 19309241

[B62] Baron F Cotransplantation of mesenchymal stem cells might prevent death from graft-versus-host disease (GVHD) without abrogating graft-versus-tumor effects after HLA-mismatched allogeneic transplantation following nonmyeloablative conditioning Biology of blood and marrow transplantation: journal of the American Society for Blood and Marrow Transplantation 2010 16 6 838 47 20109568 10.1016/j.bbmt.2010.01.011

[B63] McSweeney PA Hematopoietic cell transplantation in older patients with hematologic malignancies: replacing high-dose cytotoxic therapy with graft-versus-tumor effects Blood 2001 97 11 3390 400 10.1182/blood.V97.11.3390 11369628

[B64] Ning H *he correlation between cotransplantation of mesenchymal stem cells and higher recurrence rate in hematologic malignancy patients: outcome of a pilot clinical study*. Leukemia: official journal of the Leukemia Society of America Leukemia Research Fund, U.K 2008 22 3 593 599 10.1038/sj.leu.2405090 18185520

[B65] Behre G *Reply to 'The correlation between cotransplantation of mesenchymal stem cells and higher recurrence rates in hematologic malignancy patients: outcome of a pilot clinical study' by Ning* et al. Leukemia: official journal of the Leukemia Society of America Leukemia Research Fund, U.K 2009 23 1 178 author reply 179-80 10.1038/leu.2008.150 18548101

[B66] Herrmann J Lerman A The Endothelium - the Cardiovascular Health Barometer Herz 2008 33 5 343 353 10.1007/s00059-008-3088-2 18773154

[B67] Hamel E Perivascular nerves and the regulation of cerebrovascular tone J Appl Physiol 2006 100 3 1059 64 16467392 10.1152/japplphysiol.00954.2005

[B68] Saenzde Tejada I Pathophysiology of erectile dysfunction J Sex Med 2005 2 1 26 39 10.1111/j.1743-6109.2005.20103.x 16422902

[B69] Provis JM Anatomy and development of the macula: specialisation and the vulnerability to macular degeneration Clin Exp Optom 2005 88 5 269 81 10.1111/j.1444-0938.2005.tb06711.x 16255686

[B70] Izikki M Role for dysregulated endothelium- derived FGF2 signaling in progression of pulmonary hypertension Rev Mal Respir 2008 25 9 1192 10.1016/S0761-8425(08)75061-7

[B71] Basak GW Human embryonic stem cells hemangioblast express HLA-antigens J Transl Med 2009 7 27 10.1186/1479-5876-7-27 19386101 PMC2680830

[B72] Peichev M Expression of VEGFR-2 and AC133 by circulating human CD34(+) cells identifies a population of functional endothelial precursors Blood 2000 95 3 952 8 10648408

[B73] Foteinos G Rapid endothelial turnover in atherosclerosis-prone areas coincides with stem cell repair in apolipoprotein E-deficient mice Circulation 2008 117 14 1856 63 10.1161/CIRCULATIONAHA.107.746008 18378610

[B74] Werner N Intravenous transfusion of endothelial progenitor cells reduces neointima formation after vascular injury Circ Res 2003 93 2 e17 24 10.1161/01.RES.0000083812.30141.74 12829619

[B75] Wassmann S Improvement of endothelial function by systemic transfusion of vascular progenitor cells Circ Res 2006 99 8 e74 83 10.1161/01.RES.0000246095.90247.d4 16990568

[B76] Gartner S Kaplan HS Long-term culture of human bone marrow cells Proc Natl Acad Sci USA 1980 77 8 4756 9 10.1073/pnas.77.8.4756 6933522 PMC349925

[B77] Al-Lebban ZS Long-term bone marrow culture systems: normal and cyclic hematopoietic dogs Canadian Journal of Veterinary Research 1987 51 2 162 168 3607647 PMC1255296

[B78] Bagby GC Jr A monokine regulates colony-stimulating activity production by vascular endothelial cells Blood 1983 62 3 663 8 6603879

[B79] Quesenberry PJ Gimbrone MA Jr Vascular endothelium as a regulator of granulopoiesis: production of colony-stimulating activity by cultured human endothelial cells Blood 1980 56 6 1060 7 6969095

[B80] Gerson SL Friedman HM Cines DB Viral infection of vascular endothelial cells alters production of colony-stimulating activity The Journal of clinical investigation 1985 76 4 1382 90 10.1172/JCI112114 2414319 PMC424082

[B81] Tavassoli M Structure and function of sinusoidal endothelium of bone marrow Progress in clinical and biological research 1981 59B 249 256 7279943

[B82] Soda R Tavassoli M Mapping of the bone marrow sinus endothelium with lectins and glycosylated ferritins: identification of differentiated microdomains and their functional significance Journal of ultrastructure research 1983 84 3 299 310 10.1016/S0022-5320(83)80009-4 6655780

[B83] Soda R Tavassoli M Modulation of WGA binding sites on marrow sinus endothelium in state of stimulated erythropoiesis: a possible mechanism regulating the rate of cell egress Journal of ultrastructure research 1984 87 3 242 51 10.1016/S0022-5320(84)80063-5 6544873

[B84] Irie S Tavassoli M Structural features of isolated, fractionated bone marrow endothelium compared to sinus endothelium in situ Scanning electron microscopy 1986 2 615 619 3797999

[B85] Tavassoli M Structural alterations of marrow during inflammation Blood cells 1987 13 1–2 251 61 3311221

[B86] Jalili A Fifth complement cascade protein (C5) cleavage fragments disrupt the SDF-1/CXCR4 axis: further evidence that innate immunity orchestrates the mobilization of hematopoietic stem/progenitor cells Experimental hematology 2010 38 4 321 32 10.1016/j.exphem.2010.02.002 20153802 PMC2884222

[B87] Jalili A Complement C1q enhances homing-related responses of hematopoietic stem/progenitor cells Transfusion 2010 50 9 2002 10 10.1111/j.1537-2995.2010.02664.x 20456695 PMC2974804

[B88] Knudtzon S Mortensen BT Growth stimulation of human bone marrow cells in agar culture by vascular cells Blood 1975 46 6 937 43 1081892

[B89] Munker R Recombinant human TNF induces production of granulocyte-monocyte colony-stimulating factor Nature 1986 323 6083 79 82 10.1038/323079a0 3489188

[B90] Sieff CA Tsai S Faller DV Interleukin 1 induces cultured human endothelial cell production of granulocyte-macrophage colony-stimulating factor The Journal of clinical investigation 1987 79 1 48 51 10.1172/JCI112806 3491839 PMC423983

[B91] Ascensao JL Role of endothelial cells in human hematopoiesis: modulation of mixed colony growth in vitro Blood 1984 63 3 553 8 6607755

[B92] Sieff CA Niemeyer CM Faller DV The production of hematopoietic growth factors by endothelial accessory cells Blood cells 1987 13 1–2 65 74 3499195

[B93] Malone DG Production of granulocyte-macrophage colony-stimulating factor by primary cultures of unstimulated rat microvascular endothelial cells Blood 1988 71 3 684 9 3278753

[B94] Galelli A Stimulation of human endothelial cells by synthetic muramyl peptides: production of colony-stimulating activity (CSA) Experimental hematology 1985 13 11 1157 63 3877645

[B95] Segal GM McCall E Bagby GC Jr Erythroid burst-promoting activity produced by interleukin-1-stimulated endothelial cells is granulocyte-macrophage colony-stimulating factor Blood 1988 72 4 1364 7 3048443

[B96] Fibbe WE nterleukin 1 and poly(rI).poly(rC) induce production of granulocyte CSF, macrophage CSF, and granulocyte-macrophage CSF by human endothelial cells Experimental hematology 1989 17 3 229 234 2465167

[B97] Tanaka M The generation of macrophages from precursor cells incubated with brain endothelial cells–a release of CSF-1 like factor from endothelial cells The Tohoku journal of experimental medicine 1993 171 3 211 20 10.1620/tjem.171.211 8160177

[B98] Broudy VC Human umbilical vein endothelial cells display high-affinity c-kit receptors and produce a soluble form of the c-kit receptor Blood 1994 83 8 2145 52 7512842

[B99] Netelenbos T Differences in sulfation patterns of heparan sulfate derived from human bone marrow and umbilical vein endothelial cells Experimental hematology 2001 29 7 884 93 10.1016/S0301-472X(01)00653-1 11438211

[B100] Yamaguchi H Umbilical vein endothelial cells are an important source of c-kit and stem cell factor which regulate the proliferation of haemopoietic progenitor cells British journal of haematology 1996 94 4 606 11 10.1046/j.1365-2141.1996.d01-1855.x 8826881

[B101] Broudy VC Interleukin 1 stimulates human endothelial cells to produce granulocyte-macrophage colony-stimulating factor and granulocyte colony-stimulating factor Journal of immunology 1987 139 2 464 8 3298430

[B102] Suen Y Regulation of interleukin-11 protein and mRNA expression in neonatal and adult fibroblasts and endothelial cells Blood 1994 84 12 4125 34 7527667

[B103] Aggarwal R Hematopoietic stem cells: transcriptional regulation, ex vivo expansion and clinical application Current molecular medicine 2012 12 1 34 49 10.2174/156652412798376125 22082480 PMC3286491

[B104] Kita K Cord blood-derived hematopoietic stem/progenitor cells: current challenges in engraftment, infection, and ex vivo expansion Stem cells international 2011 2011 276193 21603139 10.4061/2011/276193 PMC3096303

[B105] Dahlberg A Delaney C Bernstein ID Ex vivo expansion of human hematopoietic stem and progenitor cells Blood 2011 117 23 6083 90 10.1182/blood-2011-01-283606 21436068 PMC3122936

[B106] Alcorn MJ Holyoake TL Ex vivo expansion of haemopoietic progenitor cells Blood reviews 1996 10 3 167 76 10.1016/S0268-960X(96)90023-5 8932829

[B107] Torok-Storb B Dissecting the marrow microenvironment Annals of the New York Academy of Sciences 1999 872 164 170 10.1111/j.1749-6632.1999.tb08461.x 10372119

[B108] Davis TA Porcine brain microvascular endothelial cells support the in vitro expansion of human primitive hematopoietic bone marrow progenitor cells with a high replating potential: requirement for cell-to-cell interactions and colony-stimulating factors Blood 1995 85 7 1751 61 7535587

[B109] Chute JP A comparative study of the cell cycle status and primitive cell adhesion molecule profile of human CD34+ cells cultured in stroma-free versus porcine microvascular endothelial cell cultures Experimental hematology 1999 27 2 370 9 10.1016/S0301-472X(98)00004-6 10029177

[B110] Rafii S Human bone marrow microvascular endothelial cells support long-term proliferation and differentiation of myeloid and megakaryocytic progenitors Blood 1995 86 9 3353 63 7579438

[B111] Brandt JE Bone marrow repopulation by human marrow stem cells after long-term expansion culture on a porcine endothelial cell line Experimental hematology 1998 26 10 950 61 9728930

[B112] Davis TA Conditioned medium from primary porcine endothelial cells alone promotes the growth of primitive human haematopoietic progenitor cells with a high replating potential: evidence for a novel early haematopoietic activity Cytokine 1997 9 4 263 75 10.1006/cyto.1996.0163 9112335

[B113] Brandt JE Ex vivo expansion of autologous bone marrow CD34(+) cells with porcine microvascular endothelial cells results in a graft capable of rescuing lethally irradiated baboons Blood 1999 94 1 106 13 10381503

[B114] Salter AB Endothelial progenitor cell infusion induces hematopoietic stem cell reconstitution in vivo Blood 2009 113 9 2104 7 10.1182/blood-2008-06-162941 19141867 PMC2651019

[B115] Chute JP Transplantation of vascular endothelial cells mediates the hematopoietic recovery and survival of lethally irradiated mice Blood 2007 109 6 2365 72 10.1182/blood-2006-05-022640 17095624 PMC1852197

[B116] Montfort MJ Adult blood vessels restore host hematopoiesis following lethal irradiation Experimental hematology 2002 30 8 950 6 10.1016/S0301-472X(02)00813-5 12160847

[B117] Li B Endothelial cells mediate the regeneration of hematopoietic stem cells Stem cell research 2010 4 1 17 24 10.1016/j.scr.2009.08.001 19720572 PMC2938793

[B118] Hollenberg CH Vost A Regulation of DNA synthesis in fat cells and stromal elements from rat adipose tissue J Clin Invest 1969 47 11 2485 98 4304653 10.1172/JCI105930 PMC297413

[B119] Gaben-Cogneville AM Differentiation under the control of insulin of rat preadipocytes in primary culture Isolation of homogeneous cellular fractions by gradient centrifugation. Biochim Biophys Acta 1983 762 3 437 44 10.1016/0167-4889(83)90009-5 6405801

[B120] Zuk PA Multilineage cells from human adipose tissue: implications for cell-based therapies Tissue Eng 2001 7 2 211 28 10.1089/107632701300062859 11304456

[B121] Zuk PA Human adipose tissue is a source of multipotent stem cells Mol Biol Cell 2002 13 12 4279 95 10.1091/mbc.E02-02-0105 12475952 PMC138633

[B122] Faustini M onexpanded mesenchymal stem cells for regenerative medicine: yield in stromal vascular fraction from adipose tissues Tissue engineering. Part C, Methods 2010 16 6 1515 21 10.1089/ten.tec.2010.0214 20486782

[B123] Rodriguez JP Autologous stromal vascular fraction therapy for rheumatoid arthritis: rationale and clinical safety International archives of medicine 2012 5 5 10.1186/1755-7682-5-5 22313603 PMC3296619

[B124] Auxenfans C Adipose-derived stem cells (ASCs) as a source of endothelial cells in the reconstruction of endothelialized skin equivalents Journal of tissue engineering and regenerative medicine 2012 6 7 512 8 10.1002/term.454 21755603

[B125] Martinez-Estrada OM Human adipose tissue as a source of Flk-1+ cells: new method of differentiation and expansion Cardiovascular research 2005 65 2 328 33 10.1016/j.cardiores.2004.11.015 15639471

[B126] Miranville A Improvement of postnatal neovascularization by human adipose tissue-derived stem cells Circulation 2004 110 3 349 55 10.1161/01.CIR.0000135466.16823.D0 15238461

[B127] Sumi M Transplantation of adipose stromal cells, but not mature adipocytes, augments ischemia-induced angiogenesis Life sciences 2007 80 6 559 65 10.1016/j.lfs.2006.10.020 17157325

[B128] Planat-Benard V Plasticity of human adipose lineage cells toward endothelial cells: physiological and therapeutic perspectives Circulation 2004 109 5 656 63 10.1161/01.CIR.0000114522.38265.61 14734516

[B129] Nakagami H Novel autologous cell therapy in ischemic limb disease through growth factor secretion by cultured adipose tissue-derived stromal cells Arteriosclerosis, thrombosis, and vascular biology 2005 25 12 2542 7 10.1161/01.ATV.0000190701.92007.6d 16224047

[B130] Rehman J Secretion of angiogenic and antiapoptotic factors by human adipose stromal cells Circulation 2004 109 10 1292 8 10.1161/01.CIR.0000121425.42966.F1 14993122

[B131] Cai L Suppression of hepatocyte growth factor production impairs the ability of adipose-derived stem cells to promote ischemic tissue revascularization Stem Cells 2007 25 12 3234 43 10.1634/stemcells.2007-0388 17901400

[B132] Sumi M Transplantation of adipose stromal cells, but not mature adipocytes, augments ischemia-induced angiogenesis Life Sci 2007 80 6 559 65 10.1016/j.lfs.2006.10.020 17157325

[B133] Black LL Effect of adipose-derived mesenchymal stem and regenerative cells on lameness in dogs with chronic osteoarthritis of the coxofemoral joints: a randomized, double-blinded, multicenter, controlled trial Vet Ther 2007 8 4 272 84 18183546

[B134] Black LL Effect of intraarticular injection of autologous adipose-derived mesenchymal stem and regenerative cells on clinical signs of chronic osteoarthritis of the elbow joint in dogs Vet Ther 2008 9 3 192 200 19003780

[B135] Vet-Stem http://www.vet-stem.com

[B136] Black LL Effect of adipose-derived mesenchymal stem and regenerative cells on lameness in dogs with chronic osteoarthritis of the coxofemoral joints: a randomized, double-blinded, multicenter, controlled trial Veterinary therapeutics: research in applied veterinary medicine 2007 8 4 272 284 18183546

[B137] Lin K Characterization of adipose tissue-derived cells isolated with the Celution system Cytotherapy 2008 10 4 417 26 10.1080/14653240801982979 18574774

[B138] Tissue genesis cell isolation system http://www.tissuegenesis.com/TGI%201000%20Product%20Brochure.pdf

[B139] Riordan NH Non-expanded adipose stromal vascular fraction cell therapy for multiple sclerosis Journal of translational medicine 2009 7 29 10.1186/1479-5876-7-29 19393041 PMC2679713

[B140] Ichim TE Autologous stromal vascular fraction cells: a tool for facilitating tolerance in rheumatic disease Cellular immunology 2010 264 1 7 17 10.1016/j.cellimm.2010.04.002 20537320

[B141] Hang-Fu L Marmolya G Feiglin DH Liposuction fat-fillant implant for breast augmentation and reconstruction Aesthetic Plast Surg 1995 19 5 427 37 10.1007/BF00453876 8526159

[B142] Klein AW Skin filling. Collagen and other injectables of the skin Dermatol Clin 2001 19 3 491 508 ix 10.1016/S0733-8635(05)70290-4 11599406

